# Neuropsychological approach to subjective cognitive complaints in cognitively unimpaired older people: A systematic review

**DOI:** 10.1002/gps.5728

**Published:** 2022-05-24

**Authors:** Lucía Pérez‐Blanco, Dolores Rodríguez‐Salgado

**Affiliations:** ^1^ Department of Developmental Psychology and Education Faculty of Psychology Universidade de Santiago de Compostela Santiago of Compostela Spain; ^2^ Department of Clinical Psychology and Psychobiology Faculty of Psychology Universidade de Santiago de Compostela Santiago of Compostela Spain

**Keywords:** aging, cognitive restructuring, cognitive stimulation, group discussion, physical exercise, psychoeducation, subjective cognitive complaints, systematic review

## Abstract

**Objective:**

A systemized approach to subjective cognitive complaints (SCCs) in elderly people is needed owing to the high prevalence of such complaints and their impact on the psychosocial well‐being of those affected. The aim of this study was to carry out a systematic review of the characteristics and effectiveness of intervention programmes that use a neuropsychological approach to target SCCs in cognitively unimpaired older people and that are tested in randomized controlled trials.

**Methods:**

The search included a time‐unlimited query of Scopus, PsycInfo and Medline, yielding 215 articles, of which only 7 met the inclusion/exclusion criteria.

**Results:**

The number of intervention programmes was very limited (11 interventions), but diverse, with cognitive stimulation, physical exercise, psychoeducation and cognitive restructuring all used to address SCCs.

**Conclusions:**

Interventions including only cognitive stimulation were not effective in reducing SCCs, but interventions including cognitive stimulation and psychoeducation, physical exercise, and group sessions and discussions reinforced by the therapist were effective.

## Key points

- Cognitive failures could affect negatively on the emotional and psychosocial well‐being.
- Cognitive training in combination with psychoeducation and psychical exercise showed the strongest evidence in improving subjective and objective cognitive functioning.
- Initial guidelines for designing successful interventions to target SCCs in cognitively unimpaired older people are provided.
- The review has implications for promotion of healthy cognitive aging.

## 1 INTRODUCTION

Cognitive failure, particularly regarding memory, is one of the major concerns of older people. The subjective experience and manifestation of this failure take the form of complaints, one of the main reasons why older people consult their general practitioners.^1^ The presence of cognitive complaints is considered one of the first symptoms of cognitive impairment^2^ and is a necessary criterion for the diagnosis of Mild Cognitive Impairment^3^ or the Neurocognitive Disorders included in the DSM‐5^4^ Although cognitive complaints are not always associated with these clinical conditions^5^, ^6^ and have been suggested to form part of the normal aging process,^7^ recent research suggests that the presence of cognitive complaints is associated with psychosocial risk factors^8^ such as symptoms of depression^7^ and anxiety.^9^ Furthermore, cognitive complaints may affect the daily occupational and social sphere^10^ and become an important secondary stress factor. This stress may increase the perception of memory lapses in older people, which in turn may increase the level of strain and further perceived lapses, resulting in a vicious cycle of stress.^11^, ^12^ These data support the relevance of addressing them to lessen their impact.

The term Subjective Cognitive Complaints (SCCs) has been coined to refer to self‐reported or third party‐reported cognitive disturbances, in the absence of objective cognitive impairment and underlying pathological conditions.^13^ The information provided by a third party generally concerns the perception of a problem and is not strictly subjective, although it is likely to be influenced by the individual's complaining behavior.^14^

Pharmacological interventions have shown effectiveness to improve cognitive function in patients with objective cognitive impairment,^15^, ^16^, ^17^ but not to reduce SCCs in healthy older adults.^18^ Anyway, even if pharmacological treatment were effective to reduce them, due to its potential side effects, non‐pharmacological interventions should be provided as the first choice for healthy individuals with SCCs. Non‐pharmacological intervention programmes based on cognitive training^19^, ^20^, ^21^ or moderate aerobic exercise^22^ have been implemented in order to address cognitive complaints and the associated distress. However, the effectiveness of such programmes is not clear, as neither memory training or cognitive stimulation appear to reduce cognitive complaints, with other strategies such as psychoeducation or cognitive restructuring being more effective.^11^, ^13^

We are aware of the existence of previous systematic reviews that have analyzed the effectiveness of non‐pharmacological interventions to reduce SCCs.^11^, ^13^, ^18^ However, the present systematic review adds to the previous literature by posing a broader research question that also includes analyzing the main characteristics of the components of the interventions. Moreover, it differs from previous reviews in research methodology, regards to the keywords, focusing on both SCCs and related psychosocial factors. It also distinguish regard to the inclusion criteria, considering only those studies that include a group of people over 60 years of age with SCCs and that use measures of subjective cognitive functioning. Therefore, the present systematic literature review aimed to clarify the state of knowledge on neuropsychological interventions aimed at SCCs in cognitively unimpaired older people, by (a) determining the characteristics of neuropsychological intervention programmes aimed at managing SCCs and that are tested in randomized controlled trials. Neuropsychological intervention is defined as that including not only a cognitive approach but also a functional and psychosocial approach, and (b) summarizing the results of these interventions in terms of their impact on subjective cognitive functioning and/or objective performance.

## 2 METHOD

### 2.1 PICOD framework

Research questions were raised using the PICOD framework, where P (Participants/Population) represents cognitively unimpaired older people with subjective cognitive complaints (SCCs); I (Intervention) = neuropsychological interventions were defined as cognitive stimulation, psychoeducation, cognitive restructuring and physical exercise; C (Comparison) = any control group (i.e. social participation); O (Outcome) = improving subjective and objective cognitive functioning impact; D (design) = randomized controlled trials studies.

### 2.2 Search strategy

The systematic review was conducted according to the recommendations of the PRISMA 2020 declaration (*Preferred Reporting Items for Systematic Review and Meta‐analyses statement*).^23^

The search strategy included querying the Scopus, PsycInfo and Medline databases, without any time limit, including studies published up to September 2021. The following keywords were used: (subjective cognitive complaints OR cognitive complaints) AND (approach OR training OR intervention OR treatment OR therapy) AND (non‐pharmacological intervention OR cognitive intervention OR neuropsychological intervention) AND (anxiety OR depression OR perceived OR self‐report OR self‐efficacy OR confidence OR complainer OR subjective OR belief OR beliefs). The title was screened first, followed by abstracts and full article texts. References were selected using RefWorks, and duplicates were removed using the same software.

### 2.3 Study selection

**FIGURE 1:**
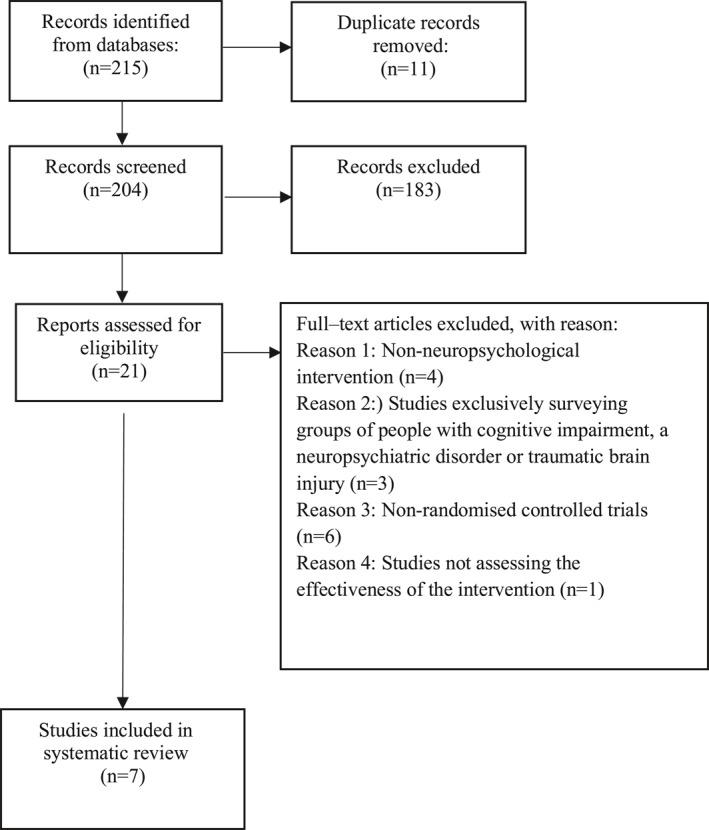
Flow diagram of the systematic review study selection process

One researcher (L.P‐B) examined titles, abstracts and full‐text articles independently. The contribution of the second author (D.R‐S.) was requested when necessary. Results of the selection process are shown in Figure 1. The inclusion criteria were as follows: (a) studies with a group of people older than 60 years with SCCs; (b) randomized controlled trials; (c) studies including measurement of SCCs (i.e. answer/questionnaire); (d) studies evaluating the efficacy of non‐pharmacological interventions in SCCs; (e) articles published in peer‐reviewed journals; (f) articles published in Spanish and English languages. Exclusion criteria were as follows: (a) studies with pharmacological interventions and/or any type of intervention not defined as neuropsychological in character; (b) studies exclusively investigating people with cognitive impairment (i.e. MCI, dementia), severe neuropsychiatric disorder or traumatic brain injury; (c) studies with healthy older people without SCCs but who wish to improve their cognitive performance; (d) non‐randomized controlled trials; and (e) theoretical review.

### 2.4 Data extraction

A standardized Excel spreadsheet was compiled for each of the studies related to: study information (i.e. first author, year), the characteristics of the sample (i.e. size of sample, sex, age and years of schooling), experimental design (i.e. number of experimental groups), interventions (i.e. format, duration, type of programme and number of sessions), the measures of objective and subjective cognitive functioning (i.e. questionnaire/answer) used and the main results were examined.

### 2.5 Quality and risk‐of‐bias assessment

Review quality was assessed using the quality assessment tool for A Measurement Tool to Assess Systematic Reviews (*AMSTAR*).^24^ The tool includes 11 criteria on which quality is determined. The instrument considers 5 critical domains: description of the systematic review protocol, adequate literature search, justification of included and excluded studies, risk of bias of individual studies and application of appropriate meta‐analytical methods.^24^ The criteria were rated as either “yes”, “no”, “I can't answer” and “not applicable”. The level of the quality was deemed to be high where it had a score greater or equal to 9. Regarding risk‐of‐bias assessment, the revised Cochrane risk‐of‐bias tool for randomized trials (RoB 2) was applied.^25^ The tool rated 5 key domains related to the following: the randomization process, the allocation and adherence of the intervention, the measures used and the outcome. The criteria were rated as either “yes”, “no”, “not applicable”. Risk‐of‐bias judgment is determined as “low risk”, “under/moderate risk” and “high risk”. This process was conducted independently by one author (L.P‐B.), who requested the input of the second author (D.R‐S) when in doubts.

## 3 RESULTS

The literature search identified 204 articles after removal of duplicate citations with the RefWorks application. After the title and abstract were examined, 183 articles were excluded and the texts of the remaining 21 articles were read in full, in order to decide on eligibility according to inclusion and exclusion criteria. The main reasons for exclusion were as follows: interventions not defined as neuropsychological in nature; studies that exclusively investigated groups of individuals with cognitive impairment, severe neuropsychiatric disorder or traumatic brain injury; and non‐randomized controlled trials. Finally, 7 studies were included for systematic review about neuropsychological intervention programmes addressing SCCs in cognitively unimpaired older people. The PRISMA flow diagram is shown in Figure 1.

The main features of the studies included in the review are summarized in Table 1. Their presentation below have been organized into two main sections: one related to the general characteristics of the studies and the other to the main results of the neuropsychological approach to SCCs.

**TABLE 1:** Non‐pharmacological intervention programmes with a neuropsychological approach targeting at SCCs

Study	Sample	Intervention	Assessment	Main results	Risk‐of‐bias
Barnes et al. (2013)	**n** = 126 **Groups:** –EG1 n = 32 (63% women) Age: *M* = 74.8 (*SD* = 6.1); Years of schooling: *M* = 16.3 (*SD* = 2.1) –EG2 n = 31 (68% women); Age: *M* = 73.8 (*SD* = 5.7); Years of schooling: *M* = 15.6 (SD = 2.8) – EG3 n = 31(58% women) Age: *M* = 71.1 (SD = 5.5); Years of schooling *M* = 16.8 (SD = 2.3) –CG n = 32 (63% women) Age: *M* = 73.9 (*SD* = 6.3); Years of schooling *M* = 16.7 (*SD* = 2.2)	Format: Cognitive training: Individual computer program; Physical activity: Group (max. 12) Duration: 12 weeks Interventions in groups: –EG1 = Games to improve speed‐accuracy, visual and auditory processing + aerobic exercises –EG2 = Games to improve speed‐accuracy, visual and auditory processing + stretching and toning up –EG3 = Educational lectures + aerobic exercises –CG = Educational lectures + stretching and toning exercises Sessions: 36 (2 h/session; 3 days/week); Cognitive training = 1 h/day; Physical activity = 1 h/day	Cognitive measures –Subjective: Do you feel that your memory or thinking skills have worsened? –Objective: RAVLT, TMT, EFT, UFOV, MMSE, Other measures –Physical fitness test	–Cognitive training improves memory, performance and divided/selective attention. –Only significant in attention. –Aerobic exercise per se does not improve cognition. –EG1 = significant improvement in global cognition.	Low risk
Boa Sorte Silva et al. (2020)	**n** = 127 **Groups**: ––EG n = 63 (70% women); Age: *M* = 67.6 (*SD* = 7.5); Years of schooling: *M* = 13.3 (*SD* = 2.7) ––CG n = 64 (72% women); Age: *M* = 67.4 (*SD* = 7.2); Years of schooling *M* = 13.8 (*SD* = 3)	**Format:** Group (max. 25); max. 6 for Mind‐Motor training **Duration**: 24 weeks **Interventions in groups:** –EG = Multimodal exercise (aerobic + resistance) and Mind‐Motor training (MME) (visuospatial working memory tasks) –CG = Multimodal exercise (balance + range of motion) and breathing **Sessions:** 62 (1 h/day; 3 days/week) –EG = 45 min exercise +15 min Mind‐Motor training; –CG = 1 h. exercise	Cognitive measures –Subjective: Do you feel that your memory or thinking skills have worsened? –Objective: CBS, MMSE, MOCA, Other measures –Physical: Heart rates –Psychosocial: Borg Classification of Perceived Stress	–EG = significant improvement in visuospatial working memory.	Moderate risk: some concerns in allocation process, measures used and outcome.
Cohen‐Mansfield et al. (2015)	**n** = 44 **Groups:** – EG1 n = 15 (60% women); Age *M* = 72.8 (*SD* = 3.78) Years of schooling *M* = 14.2 (*SD* = 4.2) – EG2 n = 15 (87% women); Age: *M* = 74.4 (*SD* = 5.78) Years of schooling: *M* = 14.5 (*SD* = 4.2) – CG n = 14 (71% women); Age: *M* = 73.2 (*SD* = 5.9) years of schooling *M* = 16 (*SD* = 2.6)	**Format:** Group (N not specified) **Duration:** 10 weeks **Interventions in groups:** –EG1 = Cognitive training (ACTIVE) in memory, execution and attention. Memory strategies. Discussion; –EG2 = Emotional, physical and social health promotion workshop; –CG = Social participation **Sessions**: Not specified	Cognitive measures –Subjective: Self‐report on memory difficulties. –Objective: MMSE, GCS, Other measures – Psychosocial: UCLA, OARS, GDS	–EG1, EG2, CG = improvement of global cognitive status, and only significant improvement of memory and visuospatial ability. –EG1 = significant improvement in self‐reported memory complaints.	Moderate risk: some concerns in allocation process, adherence of intervention and the outcome.
Frankenmolen et al. (2018)	**n** = 60 **Groups:** – EG n = 31 (32% women) Age: *M* = 66.2 (*SD* = 7.3) Years of schooling: *M* = 4.5 (*SD* = 1.9) – CG n = 29 (66% women) Age: *M* = 68 (*SD* = 7.8) Years of schooling: *M* = 4.7 (*SD* = 2)	**Format**: Small group (3–5) **Duration**: 7 weeks **Follow‐up:** after to 6 months **Interventions in groups**: –EG = Psychoeducational intervention. Cognitive restructuring. Personal goal setting. Memory strategies. Discussions. –CG = Psychoeducational intervention. Setting personal goals. Digital cognitive training (COGPACK): Attention + memory. **Sessions:** 7 (1 h/day; 1 day/week)	**Cognitive measures** **–Subjective**: MCQ **–Objective**: MOCA, RBMT‐3, LLT, RAVLT, TMT, subscale of memory from WAIS‐IV **Other measures:** –**Psychosocial:** SUI, RAND‐36 –**Functional:** IADL	–Non‐significant improvement in complaints. –Significant generalization of the use of internal strategies. –Non‐significant improvement in personal goals with respect to memory.	Moderate risk: some concerns in allocation process, adherence of intervention and the outcome.
Hoogenhout et al. (2012)	**n** = 60 **Groups**: – EG n = 30; (100% women); Age *M* = 66.0 (*SD* = 4.2); Years of schooling: *M* = 4.14 (*SD* = 2.03) – CG n = 30 (100% women) Age: *M* = 66.1 (*SD* = 4.4); Years of schooling: *M* = 4.0 (*SD* = 1.9)	**Format:** Group (6–9) **Duration**: 4 weeks **Sessions**: 8 (1h30 m/session; 2 days/week) **Interventions in groups:** –EG = Psychoeducational intervention: Cognitive aging, contextual factors, compensatory behaviors. Discussion. Cognitive diary. –CG = Waiting list	**Cognitive measures** **–Subjective:** MMI **–Objective:** MMSE, MQ, ESQ, **Other measures** –**Psychosocial:** PWQ	–EG = Significant reduction in negative emotional reactions to cognition. –No significant improvement in objective cognition.	High risk in allocation process, adherence of intervention, and some concerts measures used and the outcome.
Kwok et al. (2012)	**n** = 223 **Groups:** – EG n = 112 (88% women) Age: *M* = 75.4 (*SD* = 5.82) Uneducated = 5.4% Primary = 75.7% Secondary = 18.9% –CG n = 111 (83% women); Age: *M* = 75.3 (*SD* = 5.83); Uneducated = 12.5%; Primary = 64.3%; Secondary = 23.2%	**Format:** Group (N no specified) **Duration:** 12 weeks **Follow‐up**: after to 9 months **Interventions in groups:** –EG = Cognitive training: Memory, attention and reasoning. –CG = Attendance at health‐related educational conferences. **Sessions:** 12 (1h30 m/session; 1 day/week)	**Cognitive measures:** **–Subjective**: CMSS **–Objective:** CMMSE, CDRS	–EG = Improved memory and SCCs of people with low educational attainment.	Moderate risk: some concerns in allocation process, adherence of intervention and the outcome.
Pereira‐Morales et al. (2017)	**n** = 40 **Groups:** – EG1 n = 17 (88% women) Age: *M* = 64.5 (*SD* = 6.8) Years of schooling: *M* = 10.5 (*SD* = 4) – EG2 n = 12 (92% women) Age: *M* = 69.3 (*SD* = 4.8) Years of schooling:: *M* = 13.2 (*SD* = 3.1) – CG n = 11 (91% women) Age: *M* = 65.6 (*SD* *=* 7.2); Years of schooling: *M* = 13.3 (*SD* = 3.2)	**Format:** Group (N no specified): Computerized Cognitive training (CCT): Individual **Duration:** 8 weeks **Interventions in groups:** –EG1 = Integrated Programme of psychostimulation: CCT (domains: Attention; memory, executive, and orientation) (60 min) + traditional Cognitive training (attention, memory, executive, and orientation) (60 min) + Metamemory training. Relaxing. Reflexion. Feedback (30 min) –EG2 = CCT (domains: Attention; memory, executive, and orientation) –CG = Read a leaflet with recommendations to reduce the risk of MCI. **Sessions:** 32; (1h30 m/session; 4 days/week)	**Cognitive measures:** **–Subjective**: SMCQ **–Objective:** MMSE, Wechsler memory and attention subscales (digits, Span digits, and symbol search). Clock test, Trail Making test, SCWT, **Other measures:** –**Psychosocial:** GDS, STAI	–IPP = Non‐significant improvement in SCCs and in the specific cognitive domains trained –EG1 = Significant improvement in global cognition and anxious symptoms.	Moderate risk: some concerns in allocation process, adherence of intervention and the outcome.

*Note*: Bold is to highlight the different types of content.

Abbreviations: ACTIVE, Advanced Cognitive Training for Independent and Vital Elderly; CBS, Cambridge Cognitive Computerized Cognitive Brain Science Battery; CG, control group; CMMSE, Chinese version of Mini‐mental state examination; CMSS, Chinese Memory Symptom Scale; CST, Concept Shifting Test; EFT, Eriksen Flanker Test; EG, experimental group; ESQ, Executive functioning and Speed Quotient; GCS, Global Cognitive Score; GDS, Geriatric Depression Scale; IADL, Instrumental Activities of Daily Living Scale; LDST, Letter Digit Substitution Test; LLT, Location Learning Test; MMI, Maastricht Metacognition Inventory; MMSE, Mini Mental State Examination; MOCA, Montreal Cognitive Assessment; MQ, Memory Quotient; OARS, Older American Resources and Services; PWQ, Psychological Well‐being Quotient; RAND‐36, Health Survey; RAVLT, Rey Auditory Verbal Learning Test; RBMT‐3, Rivermead Behavioral Memory Test; SCWT, Stroop Color‐Word Test; SMCQ, Subjective Memory Complaints Questionnaire; STAI, State‐Trait Anxiety Inventory; SUI, Strategy Use Inventory; TMT, Trail Making Test; UCLA, UCLA Loneliness scale; UFOV, Useful Field of View; VVLT, Visual Verbal Learning Test.

### 3.1 General characteristics of the studies included

The sample size of the studies included in the review ranged from 40 to a maximum of 223 participants. The mean age of the participants was 64.33 years (*SD* = 7.24) in the experimental group and 69.92 years (*SD* = 11.85) in the control group. Two studies (28%) were conducted in North America,^26^, ^27^ two (28%) in Asia,^19^, ^20^ two (28%) in Europe,^10^, ^28^ and one (16%) in South America.^21^ The majority of studies recruited participants from the community, except for three studies that recruited them in long‐term care centers.^19^, ^20^, ^21^ In relation to educational level, the mean number of years of schooling was 12.3 (*SD* = 4.33) in the experimental group and 11.42 (*SD* = 5.1) in the control group, although one study provided data according to educational level, for both the experimental group (no education = 5.4%; primary = 75.7%; secondary = 18.9%) and the control group (no education = 12.5%; primary = 64.3%; secondary = 23.2%).

In the 7 studies reviewed, 11 different interventions were identified as having been carried out. Most of the interventions had a group format,^10^, ^19^, ^20^, ^21^, ^26^, ^27^, ^28^ with the number of participants in the group ranging from a minimum of 3^10^ to a maximum of 25.^27^ The interventions had an individual format in only two cases.^21^, ^28^ The duration of the programmes was not very uniform, ranging from 4 weeks^28^ to 24 weeks^27^ and only two studies included follow‐ups, after 6^10^ or 9 months.^20^

The number of sessions in the programmes ranged from a minimum of 7^10^ to a maximum of 62.^27^ The duration of the sessions varied between 1 h^10^, ^26^, ^27^ and 1 h 30 min.^20^, ^21^, ^28^ In addition, the number of sessions per week ranged from 1^10^, ^20^ to 4.^21^

Regarding the type of programmes or strategies used in the intervention, 4 categories were identified. The first category includes those interventions referring to specific cognitive training.^19^, ^20^, ^21^ The second category includes those studies in which the interventions combine cognitive training and physical activity.^26^, ^27^ The third category includes interventions focusing on psychoeducation and health promotion.^19^, ^27^ Finally, the fourth category combines cognitive training with cognitive restructuring and pschoeducation.^10^

Analysis of the strategies used shows that cognitive training aims to optimize and maintain the overall cognitive state through tasks that involve training in strategies and skills. The strategies are mainly aimed at episodic memory training,^10^, ^19^, ^20^, ^21^, ^26^, ^27^ but also consider visuospatial ability,^27^ reasoning,^20^ processing speed,^19^, ^26^ attention^10^, ^20^, ^21^, ^26^ and executive function.^21^, ^26^ The cognitive Mind‐Motor training used in one study^27^ specifically focuses on improving visuospatial episodic memory, based on an activity that depends on the number of steps and the order and direction of the feet. Interventions focusing on physical activity always include aerobic exercises^26^, ^27^ and the sessions end with breathing and relaxation exercises, except for one that only included stretching and toning exercises.^26^ Finally, psychoeducational intervention strategies and cognitive restructuring strategies both aim to (a) raise awareness about the cognitive and functional aging process, contextual factors, compensatory strategies and behavior,^10^, ^28^ (b) change beliefs and attitudes related to memory and establish personal expectations about memory, (c) educate participants in affective‐emotional health and inform them about neurodegenerative diseases typical of aging and the associated problems^10^, ^19^, ^28^ and (d) educate the participants in physical and social health.^14^

The following methods were used to implement the interventions: digital cognitive training programmes^21^, ^26^; a grid carpet (2.5 × 1 m) for Mind‐Motor training^27^; and lecture sessions with PowerPoint presentations, in cognitive training workshops^19^, ^20^ and psycho‐educational interventions.^10^, ^19^, ^28^

Finally, in relation to the cognitive measures used, for subjective assessment, a general question related to memory “*Do you feel that your memory or thinking skills have worsened?* ^26^, ^27^ or the application of different questionnaires on forgetfulness^10^, ^19^, ^20^, ^21^, ^28^ have been used. In order to assess objective performance in relation to global cognitive status, most studies used the MMSE screening test^19^, ^20^, ^21^, ^27^, ^28^ or the MoCA.^10^, ^27^ For assessment of specific cognitive domains, different neuropsychological tests, such as the Trail Making Test^10^, ^21^, ^26^ and the Wechsler memory and attention subscales,^21^ have been used.

### 3.2 Main results of the neuropsychological approach to cognitive complaints

In general, the results regarding neuropsychological intervention in SCCs indicate an improvement in cognitive functioning at the subjective level^10^, ^19^, ^20^, ^21^, ^28^ and also in objective performance,^19^, ^20^, ^21^, ^26^, ^27^ although the improvement is not always significant.

#### 3.2.1 Cognitive training (4 interventions)

The results of the three studies using interventions based on cognitive training showed the following: (a) only one intervention significantly reduced self‐reported memory complaints^19^; (b) three interventions improved performance in the specific cognitive domains trained, that is, memory,^19^, ^20^ visuospatial ability,^19^ conceptualization,^20^ attention, executive function/mental inhibition/flexibility and orientation (Integrated Programme of Psychostimulation)^21^; (c) only the Integrated Psychostimulation Program^21^ significantly improved global objective cognitive performance; and finally, (d) two interventions improved SCCs although not significantly: the Integrated Programme of Psychostimulation^21^ and cognitive training intervention.^20^

#### 3.2.2 Cognitive training and psychical exercise (3 interventions)

Regarding interventions based on the combination of cognitive training and physical exercise used in two studies, they have shown: (a) significant improvement in global cognitive status when combined games to improve speed‐accuracy, visual and auditory processing and aerobic exercises were used^26^; (b) significant improvement in visuospatial working memory when combined multimodal exercise and Mind‐Motor Training were used^27^; (c) improvement in different cognitive domains (memory, executive function/mental inhibition/flexibility, divided and selective attention) with cognitive training using a computer program, although the improvement was only significant for attention^26^; (d) aerobic exercise alone did not lead to cognitive improvement, either objectively or subjectively.^26^

#### 3.2.3 Psychoeducation and health promotion (2 interventions)

The results of the two studies using interventions based on psychoeducation have shown improvement in subjective and objective cognitive functioning.^19^, ^28^ In particular, they have shown that: (a) the promotion of emotional, physical and social health in older people with SCCs improved subjective well‐being and objective cognitive performance, but did not lead to a significant reduction in SCCs^19^; (b) a significant improvement in the subjective cognitive reducing negative emotions towards cognitive functioning,^28^ but not in objective cognitive functioning.

#### 3.2.4 Psychoeducation, cognitive restructuring and cognitive training (1 intervention)

The intervention based on a combination of psychoeducation, cognitive restructuring and training in mnemonic strategies, improved the subjective perception of cognitive functioning and decreased SCCs, although the changes were not statistically significant.^10^

### 3.3 Methodological quality of the included studies

The quality was deemed to be high (9 out of a maximum of 11 points, corresponding to 81.81% positive responses). Five studies had a moderate/under risk of bias (71.42%); only one study had a low level of risk (14.2%), and one was high risk (14.2%). All studies specified the eligibility criteria and used randomization. The failures were mainly related to the absence of single and/or double‐blinding of participants or professionals (performance bias),^10^, ^19^, ^20^, ^27^ and to the validity of the subjective variables^28^ and the incomplete outcome date (attrition bias)^10^, ^19^, ^20^, ^21^, ^27^, ^28^

## 4 DISCUSSION

The results of the present review study show that the number of intervention programmes that use a neuropsychological approach, in randomized controlled trials, aimed at the management of SCCs in cognitively unimpaired older people is very limited. Moreover, scientific evidence shows that studies carried out to assess intervention programmes use different designs, technical perspectives and strategies. In this regard, the following types of programmes or interventions aimed at addressing SCCs have been identified: specific cognitive training alone or combined with physical exercise, and psychoeducation and/or cognitive restructuring, alone or combined with cognitive training.

The most commonly used type of intervention is cognitive training.^19^, ^20^, ^21^ Programmes based on cognitive training, mainly memory, attention and executive function/mental inhibition/flexibility, are generally organized in group sessions of 90 min each, at least once a week, and with an average duration of 10 weeks. These intervention programmes include the ACTIVE programme, which generated a significant improvement in cognitive state, both subjectively and objectively, of people with SCCs.^19^ The aims of this program and also the Integrated Psychostimulation Program^21^ are to (a) improve objective performance (specifically memory, attention and executive function), (b) generalize the use of external and internal memory strategies and, (c) encourage social participation through discussion and feedback from the professionals involved. However, unlike the ACTIVE programme, the Integrated Psychostimulation Program did not produce any significant improvement in the subjective well‐being of people with SCCs, although it did reduce the associated symptoms of anxiety. The difference in the results of the two programmes may be related to differences in their format, which is group‐based in the case of the ACTIVE programme. This consistent with previous evidence on the personal, relational and emotional benefits of group‐based interventions.^29^, ^30^, ^31^ In this regard, Yin and colleagues confirmed that the combination of group counseling and memory training would improve emotional well‐being and learning memory performance in older adults with SCCs who reported depressive or anxious symptoms.^32^ Furthermore, although specific cognitive training by itself, without reflection or group discussion, leads to improvements in objective cognitive functioning, it does not do so at the subjective level.^21^ In this regard, Oh and colleagues showed that memory training in an individual format, through a smartphone application (the Smartphone‐based brain Anti‐aging and memory Reinforcement Training, SMART), improved cognitive performance but not reduce the feeling of subjective failure^33^

Intervention programmes combining cognitive training and physical activity have also been used to address cognitive complaints, although only in two cases,^26^, ^27^ and with programmes that are organized differently. In one case, the programme is carried out in individual sessions for cognitive activity and group sessions for physical activity (with a maximum of 12 participants), with one hour for each activity, for 3 days a week, for 12 weeks.^26^ In the other case, the physical exercise and Mind‐Motor training programme is carried out in group sessions (maximum 25 participants), each of 60 min (cognitive activity = 15 min; physical activity = 45 min), 3 times a week, for 24 weeks.^27^ In both cases, the intervention improved objective cognitive performance, but did not reduce SCCs. The improvement in objective performance seems to be independent of the time dedicated in the sessions to each of the programme components. Thus, the Mind‐Motor^27^ intervention programme, which dedicates 45 min to physical activity and 15 min to cognitive training, produced the same improvement in objective cognitive performance as the intervention that specifically dedicates 1 hour to each activity.^26^ The fact that the improvement at the objective level appears to be independent of the time dedicated to each component may be related to other characteristics of the interventions such as their duration, which is 24 weeks in one study^27^ and only 12 weeks in the other.^26^ This finding is consistent with data suggesting that to be effective, interventions should last at least 6 months.^34^ Moreover, aerobic exercise by itself, without cognitive training, did not yield any improvement in global cognitive status, unlike cognitive training alone.^26^ Consistent with this finding, research by Kamegaya and colleagues showed that the practice of aerobic physical exercise alone improved some aspects of cognitive function, although not significantly, even if maintained for 12 weeks.^35^

Regarding the two programmes that include interventions based on psychoeducation, this is aimed at working both on the understanding of complaints as part of normal aging and on training and information on their influence on daily life.^19^, ^28^ Thus, three fundamental aspects are addressed in these programmes: information on the normal cognitive and functional aging process; education in affective‐emotional health; and information on the different neurodegenerative diseases typical of old age. However, the impact of both programmes on cognitive status differed, both subjectively and objectively. Only the intervention carried out in one study^28^ produced a significant improvement in subjective well‐being by reducing negative emotional reactions to cognitive functioning, although it did not improve objective cognitive performance. This finding align with those from previous studies on SCCs exploring the role of group psychoeducation in psychological well‐being.^13^, ^14^ This difference in the impact of the two programmes on subjective cognitive state may be related to differences in their characteristics. Thus, apart from differences in the number and duration of sessions, only women were included in one of them,^28^ which may bias the generalization of the results. In addition to these differences, the programmes use different types of psychoeducational intervention strategies. Importantly, one programme^28^ encouraged maximum social participation through debate and comment on doubts about the session in discussion groups and encouraged participants to keep a diary to consolidate what has been learned, unlike the other programme.^19^

Finally, regarding the intervention that includes cognitive training, cognitive restructuring and psychoeducation, only one study^10^ combined the three techniques in a programme carried out in group sessions, each of 60 min, with a maximum of five people per group, once a week, for 7 weeks. This intervention, which emphasized training and information on aging and cognitive complaints, generated a non‐statistically significant improvement in the subjective perception of cognitive status and complaints, both by enhancing personal goals regarding memory and by changing erroneous beliefs about cognitive and functional aging. In addition, the programme generated an improvement in objective cognitive functioning by succeeding in getting participants to generalize the use of internal memory strategies, although not the use of external strategies.^10^

In conclusion, the review of the literature on non‐pharmacological interventions with a neuropsychological approach aimed at SCCs in the elderly highlights two interventions that have a significant positive impact on subjective cognitive functioning. One is the ACTIVE cognitive training programme proposed by Cohen‐Mansfield and colleagues^19^ and the other is the psychoeducational intervention tested by Hoogenhout and colleagues.^28^ Regarding their characteristics, the ACTIVE programme combines training specific cognitive areas and participation in group discussions. By contrast, the intervention by Hoogenhout and colleagues^28^ in addition to providing psychoeducation on cognitive and functional aging, favored group participation and cognitive reinforcement by keeping a diary. Both programmes probably owe their efficacy at the subjective level to the organization of group sessions for a minimum of 4 weeks and to the fact that they enhanced the social intervention of the participants. These data provide initial guidelines for designing successful interventions targeting SCCs in cognitively unimpaired older people, thereby improving the current health policy for the elderly. To our knowledge no specific programmes are usually offered to older adults without objective impairment but do have cognitive complaints, rather interventions aimed at training cognitive abilities, such as cognitive stimulation or memory training programmes.

Finally, it should be pointed out that all intervention programmes based on psychoeducation and/or cognitive restructuring reduced, although not significantly, self‐reported cognitive complaints, improving the personal and social well‐being of the elderly participants. On the other hand, specific or combined cognitive training programmes (e.g. with physical exercise, psychoeducation or cognitive restructuring) improved objective cognitive performance both globally and in the areas specifically trained.^26^, ^27^

### 4.1 Limitations

Although this systematic review provides guidelines for the neuropsychological approach to SCCs in cognitively unimpaired older people, these should be applied by considering the review findings. First, the analysis of SCCs is a recent line of research, and very few studies have been conducted to date. Moreover, we were unable to carry out a meta‐analysis because the studies included were heterogeneous in terms of methodology (e.g. therapeutic approaches, cognitive domains, diversity in measurement instruments). On the other hand, most studies have used small samples.^10^, ^20^, ^21^, ^28^ Furthermore, only two studies included long‐term follow‐up of the impact of the intervention on subjective cognitive functioning and/or objective performance.^10^, ^21^ Nevertheless, a long‐term follow up assessment it would be useful to examine whether the level of stress and perceived memory lapses decreases after maintenance of the learned cognitive, social and psychoeducational strategies. More prospective longitudinal studies are needed. Lastly, the scientific evidence regarding the efficacy of the techniques used like as the cognitive restructuring is limited and therefore results in this regards should be interpreted with caution.

### 4.2 Conclusions

This systematic review showed that interventions including only cognitive stimulation were not effective in reducing Subjective Cognitive Complaints, but interventions including cognitive stimulation and psychoeducation, physical exercise, and group sessions and discussions reinforced by the therapist were effective. Despite the limited number of studies, it provides an initial guide for designing successful interventions, which should run on the organization of group sessions for a minimum of 4 weeks and to the social intervention of the participants. Our findings may have implications for public health policies focused on promoting healthy cognitive aging, and the design of prevention and intervention programs for the early stages of cognitive impairment.

## AUTHOR CONTRIBUTIONS

Concept and design: Dolores Rodríguez‐Salgado; Lucía Pérez‐Blanco; Methodology: Lucía Pérez‐Blanco; Dolores Rodríguez‐Salgado; Acquisition, analysis or interpretation of data: Lucía Pérez‐Blanco. Writing original draft: Lucía Pérez‐Blanco; Critical revision of the manuscript for important intellectual content: Dolores Rodríguez‐Salgado; Lucía Pérez‐Blanco; Writing review & editing: Dolores Rodríguez‐Salgado, Lucía Pérez‐Blanco.

## CONFLICT OF INTEREST

The authors declare that there is no conflict of interest.

## Data Availability

The data that support the findings of this study are available from the corresponding author upon reasonable request.
